# Ischiofemoral impingement syndrome: a case report and review of literature

**DOI:** 10.1186/s13018-022-03287-y

**Published:** 2022-08-19

**Authors:** Madhan Jeyaraman, Jayanth Murugan, Nicola Maffulli, Naveen Jeyaraman, Anish G. Potty, Ashim Gupta

**Affiliations:** 1 Department of Orthopaedics, Faculty of Medicine, Dr MGR Educational and Research Institute, Sri Lalithambigai Medical College and Hospital, Chennai, Tamil Nadu 600095 India; 2 South Texas Orthopedic Research Institute (STORI Inc.), Laredo, TX 78045 USA; 3 Department of Radio Diagnosis, SRM Medical College Hospital and Research Centre, SRM Institute of Science and Technology, Chengalpattu, Tamil Nadu 603211 India; 4 Department of Musculoskeletal Disorders, School of Medicine and Surgery, University of Salerno, 84084 Fisciano, Italy; 5 San Giovanni di Dio e Ruggi D’Aragona Hospital “Clinica Orthopedica” Department, Hospital of Salerno, 84124 Salerno, Italy; 6 Centre for Sports and Exercise Medicine, Barts and The London School of Medicine and Dentistry, Queen Mary University of London, London, E1 4DG UK; 7 School of Pharmacy and Bioengineering, Keele University School of Medicine, Stoke-on-Trent, ST5 5BG UK; 8 Department of Orthopaedics, Atlas Hospitals, Tiruchirappalli, Tamil Nadu 620002 India; 9 Laredo Sports Medicine Clinic, Laredo, TX 78041 USA; 10 Future Biologics, Lawrenceville, GA 30043 USA; 11 BioIntegrate, Lawrenceville, GA 30043 USA; 12 Veterans in Pain (V.I.P.), Valencia, CA 91354 USA

**Keywords:** Ischiofemoral impingement, Quadratus femoris muscle, Arthroscopy, Lesser trochanter

## Abstract

**Introduction:**

The etiology of ischiofemoral impingement (IFI) syndrome, an unusual and uncommon form of hip pain, remains uncertain. Some patients demonstrate narrowing of the space between the ischial tuberosity and lesser trochanter from trauma or abnormal morphology of the quadratus femoris muscle. Combined clinical and imaging aid in the diagnosis.

**Case report:**

A 32-year-old female presented with a 3 years history of pain over the lower aspect of the right buttock, aggravated by movements of the right hip, and partially relieved with rest and medications. The right hip showed extreme restriction of abduction and external rotation. MRI of the right hip showed reduced ischiofemoral space and quadratus femoris space when compared to the left hip. The patient underwent endoscopic resection of the right lesser trochanter, with no recurrence of pain at 2 years.

**Conclusion:**

An unusual cause of hip pain, IFI syndrome, should be suspected when hip pain at extremes of movement is associated with signal abnormality of quadratus femoris muscle. Management is tailored to address the inciting factors that precipitated the IFI syndrome.

## Introduction

In ischiofemoral impingement (IFI) syndrome, an unusual and rare form of hip pain, there is narrowing of the space between the ischial tuberosity and lesser trochanter from trauma or abnormal morphology of the quadratus femoris muscle. IFI produces non-specific hip pain during repeated movements of the hip (extension, abduction, and external rotation) which may impair the action of the quadratus femoris muscle. IFI syndrome can be idiopathic, without any precipitating trauma, or prior hip surgeries, or associated with abnormal morphology of the quadratus femoris muscle.

Clinical examination and imaging aid in diagnosing IFI syndrome. Narrowing of quadratus femoris space and ischiofemoral space with increased ischial angle and femoral neck angle is suggestive of IFI syndrome [1]. The reliability of magnetic resonance imaging (MRI) in determining the diagnosis of IFI syndrome is unclear, as similar findings can be found in asymptomatic individuals [2]. The management of IFI is dictated by the functional needs of the patients, and the management strategy is individualized. We report a patient with IFI syndrome without any history of trauma who underwent endoscopic resection of the lesser trochanter.

## Case report

A 32-year-old woman presented with a 3 years history of pain over the lower aspect of the right buttock. With insidious onset, the dull ache radiated to the posterior aspect of the lower right thigh. The symptoms were aggravated on movements of the right hip and partially relieved with rest and medications. The patient reported no history of trauma or infection and noticed an audible and a painful clunk along with the grinding sensation in her right hip. On examination, there were no swelling, scars, or sinuses around the right hip. The patient demonstrated an audible and palpable snap in her right hip when moving the right hip. The Trendelenburg sign was negative. There were no neurovascular abnormalities. There was restriction of the terminal range of abduction and external rotation of the right hip.

**Fig. 1:**
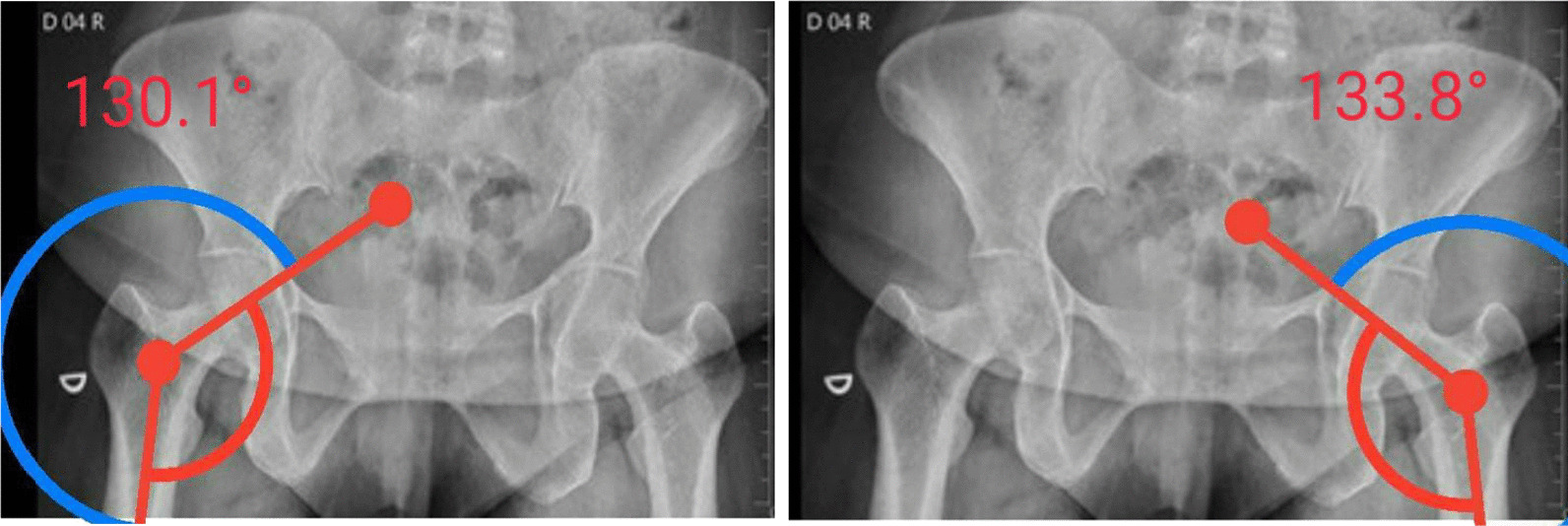
Radiograph of the pelvis with bilateral hips show the neck–shaft angle of 130.1° in right hip and 133.8° in the left hip

**Fig. 2:**
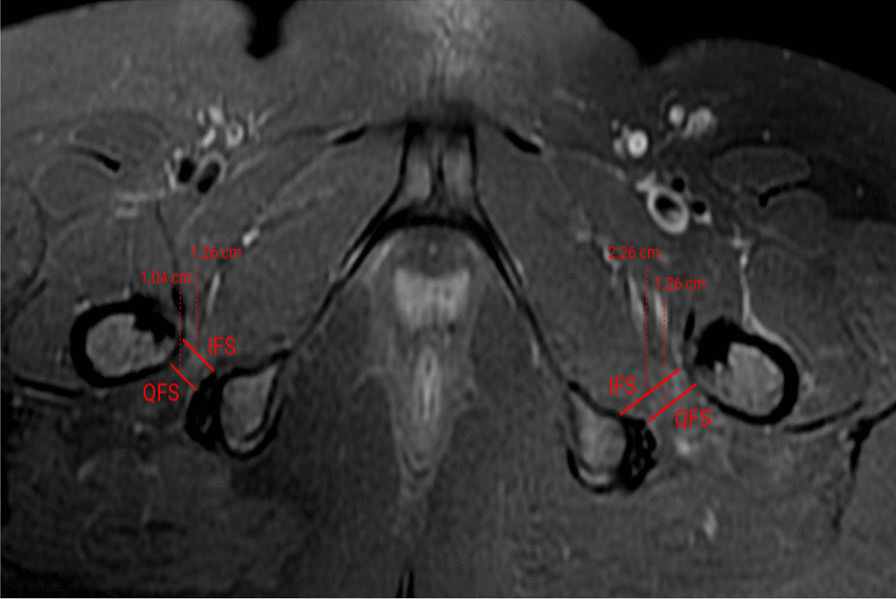
Axial T1W and PDFS MRI images, showing reduction of the right ischiofemoral space compared to the left

**Fig. 3:**
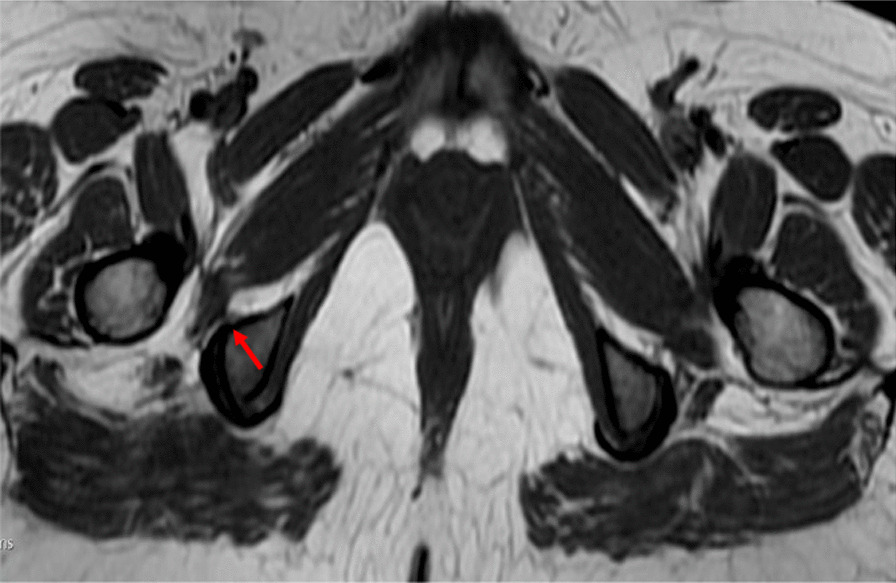
Axial T1W image: subtle fatty atrophy of the right quadratus femoris muscle (red arrow)

**Fig. 4:**
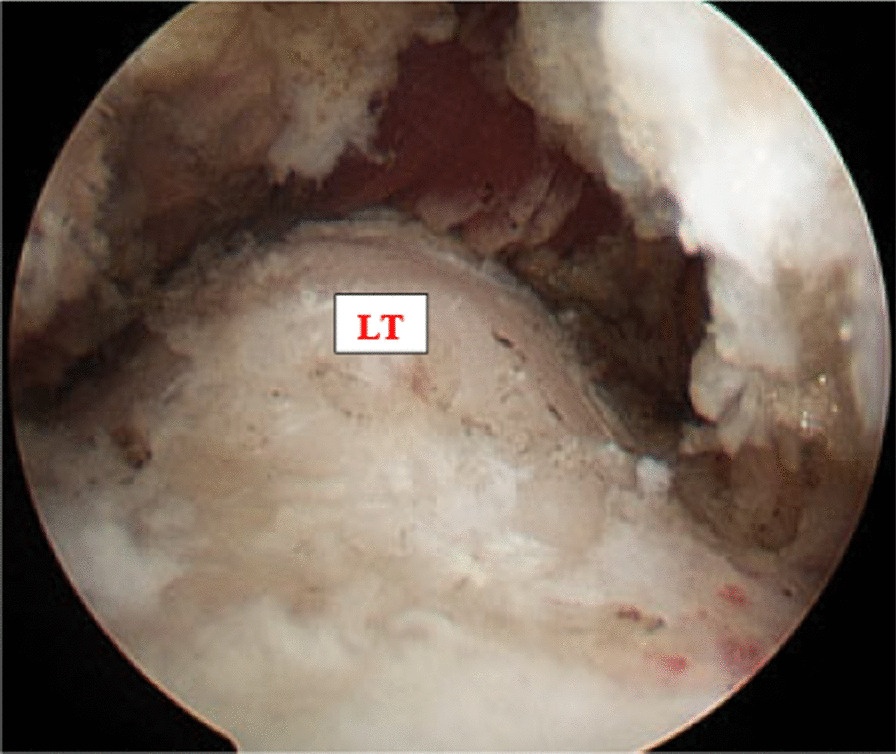
Endoscopic view of the right lesser trochanter before resection

**Fig. 5:**
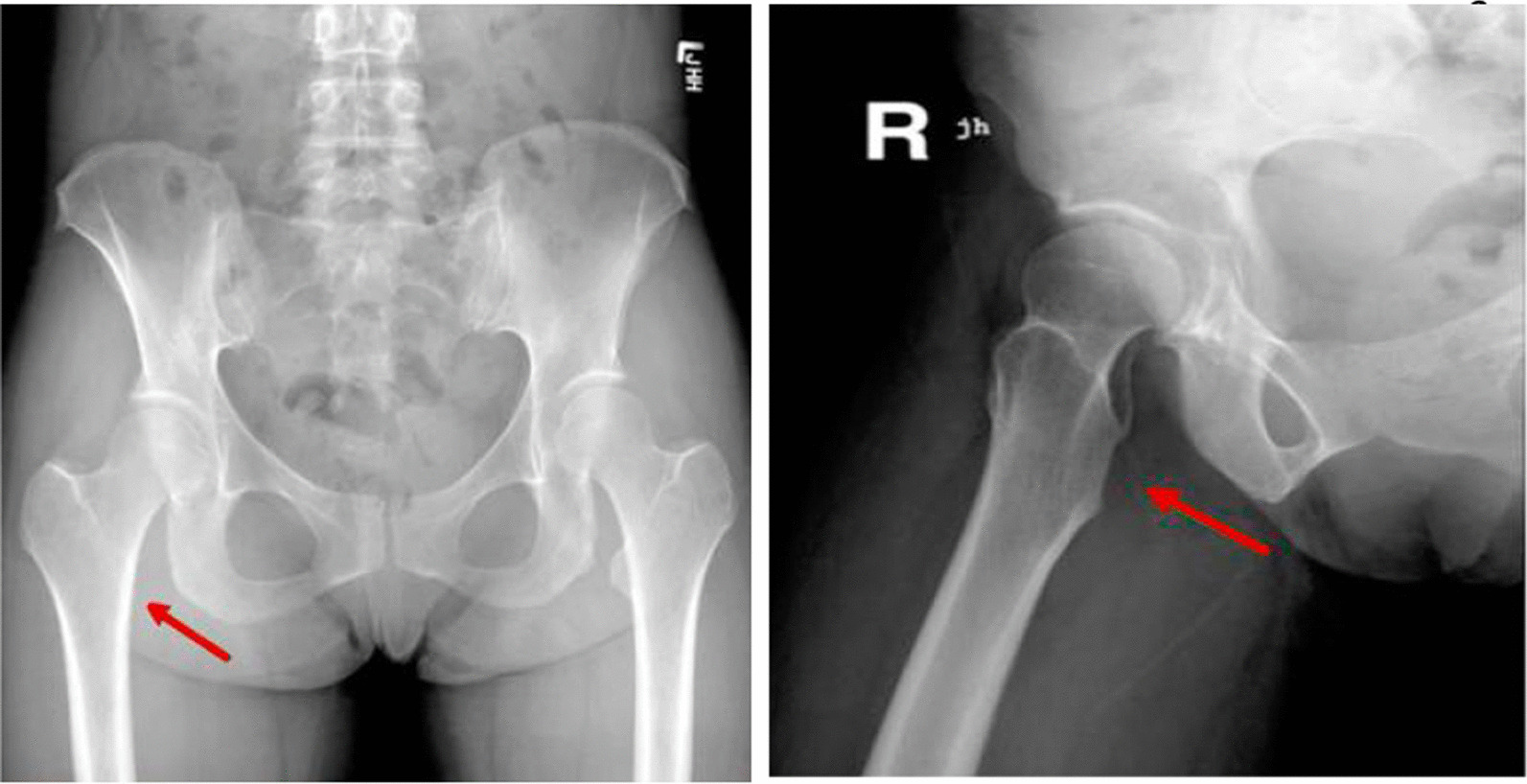
Postoperative radiograph of right hip showing resection of lesser trochanter (red arrow)

Plain radiographs of both hips showed normal acetabula bilaterally, with no evidence of pincer or cam deformity, a 35° center-edge (CE) angle bilaterally, symmetrical hip joint spaces, and valgus hips with the femoral neck–shaft angle of 130° on the right hip and 134° on the left hip (Fig. 1). The axial T1W and PDFS MRI images showed reduction of the right ischiofemoral space (12.6 mm) compared to the left ischiofemoral space (22.6 mm) (Fig. 2). The axial T1W image showed subtle fatty atrophy of the right quadratus femoris muscle when compared to the left (Fig. 3). The patient was managed surgically by endoscopic resection of the right lesser trochanter (Fig. 4). Postoperative radiographs of the right hip (anteroposterior and lateral views) confirmed the wide resection of the lesser trochanter (Fig. 5).

The patient was advised to partially weight bear for 2 weeks followed by full weight-bearing of the right lower limb. The patient experienced no pain while performing her usual activities of daily living. No heterotopic bone formation was observed. At 2 years of follow-up, the patient reported an improvement of the International Hip Outcome Tool (iHOT) score from 49 to 89 points.

## Discussion

The temporal and spatial association between hip pain and trauma or abnormal morphological changes in the quadratus femoris muscle has been previously described. In 1977, Johnson described ischiofemoral narrowing in 3 patients with unusual nature of hip pain following surgery (2 patients after total hip arthroplasty and 1 patient after proximal femoral osteotomy). In these 3 patients, symptoms were relieved by resection of the lesser trochanter [3]. An uncommon entity, IFI poses a diagnostic challenge.

IFI has been associated with the broad and shallow female pelvis, congenital posteromedial femoral position, osteochondromatosis of the hip, increased diameter of the femur at the lesser trochanter, coxa valga, coxarthrosis with superomedial migration, and malunited intertrochanteric fracture [4, 5]. IFI can result from narrowing of the quadratus femoris space from hamstrings or gluteus medius enthesopathy [6]. Abnormalities of soft tissues may lead to narrowing of the ischiofemoral space [7], and IFI may be classified as congenital, positional, and acquired [7]. IFI syndrome may result from compression of the quadratus femoris muscle from decreased ischial space, or narrowing of the space between hamstring muscles and the posteromedial aspect of the femur [8].

In IFI syndrome, the space between the ischial tuberosity and the lesser trochanter is below 20 mm, with or without a history of trauma, previous hip surgeries, or abnormal morphology of the quadratus femoris muscle. IFI is more common in females, affecting patients from the first to the seventh decade. IFI syndrome has been hypothesized to be gender-related as the ischial tuberosities are further apart in females, by rendering the ischiofemoral distance narrower [9]. Sussman et al. found an increased inter-tuberous diameter and changes in the ischial angulation in female cadavers, which account for the increased incidence of IFI syndrome in females [10]. Bilateral IFI syndrome is reported in 25% of patients [7].

Patients with IFI syndrome report non-specific posterior hip pain, with load-dependent pain on the lower buttock, with a diagnostic delay ranging from a few weeks to several years [6, 9, 11–13]. Some patients report snapping or locking sensation of the hip joint during walking: This is thought to result from the forceful bypassing of the ischium over the lesser trochanter [7, 11, 14, 15]. Ganz et al. reported a sense of instability from inadequate acetabular coverage, with hip subluxation when the lesser trochanter impinges on the ischium [16]. Patients with IFI syndrome exhibit a painless functional limb length discrepancy from compensatory abduction of the leg of the affected side to increase the distance between the lesser trochanter and the ischium [16].

At times, the pain may radiate to the knee and can be diagnosed as lumbosacral radiculopathy or sciatica. The passive motion of the affected hip to induce pain while palpating the ischium points toward IFI syndrome. Johnson described the provocative test for IFI syndrome by passively extending, adducting, and externally rotating the hip [3, 9, 17]. Passive flexion and internal rotation of the hip provokes pain by stretching the impaired quadratus femoris muscle [15]. Gómez-Hoyos et al. validated the long-stride walking test with 92% sensitivity and 82% specificity, and the passive extension and adduction test with 82% sensitivity and 85% specificity for diagnosing IFI syndrome [18]. At imaging, IFI syndrome can be diagnosed by measuring the ischiofemoral and quadratus femoris space [7].

As only few patients have been reported, there are no definitive diagnostic criteria for diagnosis, and formulating a diagnosis of IFI syndrome can be difficult [4, 19–21]. Imaging may depict heterogeneous sclerosis of the lesser trochanter and the ischium. The anteroposterior radiograph and axial proton density images may show a reduced distance between the ischium and the lesser trochanter to 0.3 cm and 0.4 cm, respectively [11]. On T2W fat-suppressed MRI, hyperintense signal from quadratus femoris muscle secondary to impingement between the ischium and prominent lesser trochanter can be evidenced [11]. Lu et al. obtained similar ischiofemoral space measurements with ultrasonography [92.0% sensitivity and 68.4% specificity] and MRI [96.0% sensitivity and 84.2% specificity] [22].

Torriani et al. observed abnormal morphology of quadratus femoris muscle in 12 IFI syndrome patients with edema in 12 (100%), partial tears in 4 (33%), and fatty infiltration in 1 (8%) [7]. Tendinopathy of the myotendinous junction of the quadratus femoris and degenerative changes in the quadratus femoris muscle have been reported [2]. In 4 patients, O’Brien et al. reported fluid collection, with either edema or hemorrhage at the myotendinous junction of the quadratus femoris muscle (1 patient with a full-thickness muscle tear and 3 patients with a partial-thickness muscle tear) [23]. Based on the literature and their own experience, Torriani et al. gave inconclusive validity for the association between the narrowing of the ischium and the lesser trochanter with the abnormal morphology of quadratus femoris muscle [7]. Singer et al. reported that the cutoff for ischiofemoral space is ≤ 15 mm (sensitivity 77%, specificity 81%, accuracy 74%) and quadratus femoris space is ≤ 10 mm (sensitivity 79%, specificity 74%, accuracy 77%) in T1W axial sequence. The STIR/T2W sequence may demonstrate edema or tears of quadratus femoris, hamstrings, or iliopsoas muscles [24].

The differential diagnoses include lumbosacral radiculopathy, sciatica, femoroacetabular impingement (FAI), IFI, iliopsoas myositis, hamstrings and gluteus medius enthesopathy, piriformis syndrome, ankylosing spondylitis, and spinal stenosis; all have to be considered in patients with pain over the lower buttock radiating to the knee [25–28].

The optimal treatment strategy is unclear, but various treatment modalities have been attempted. Management of IFI syndrome ranges from conservative management in the form of rest, activity limitation, analgesics, physical therapy, CT/US-guided steroids, anesthetics, and prolotherapy into quadratus femoris muscle, and surgical management in the form of either open or endoscopic resection of the lesser trochanter, or ischioplasty [6, 13, 29–32]. Ali et al. treated IFI syndrome conservatively with excellent functional outcome [6]. Ultrasound-guided corticosteroid injections in the quadratus femoris muscle resulted in pain relief in 2 weeks [32]. Kim et al. reported excellent functional outcomes by ultrasound-guided injection of polydeoxyribonucleotide sodium in 2 patient, possibly from destruction of nerve fibers associated with pathological neovascularity, expression of VEGF, collagen production, and fibroblast proliferation [13].

Surgery has been advocated after failure of conservative management. Johnson et al. described open resection of the lesser trochanter, and some orthopedic surgeon performed endoscopic resection of the lesser trochanter [3, 9, 33–35]. Truog et al. demonstrated a complete resolution of symptoms with an open ischioplasty 3 months postoperatively [31]. In our patient, endoscopic resection of the lesser trochanter resulted in total resolution of pain lasting for the whole duration of follow-up.

## Conclusion

An unusual cause of hip pain, IFI syndrome, should be suspected when hip pain at the extremes of movement with the signal abnormality of the quadratus femoris muscle is noted at MRI. The treatment modalities employed in these cases have to be tailored to address the inciting factors that precipitated the IFI syndrome.

## Data Availability

All the data are contained within this manuscript.

## References

[1] Akça A, Şafak KY, İliş ED, Taşdemir Z, Baysal T (2016). Ischiofemoral impingement: assessment of MRI findings and their reliability. Acta Ortop Bras.

[2] Maraş Özdemir Z, Aydıngöz Ü, Görmeli CA, Sağır KA (2015). Ischiofemoral space on mri in an asymptomatic population: normative width measurements and soft tissue signal variations. Eur Radiol.

[3] Johnson KA (1977). Impingement of the lesser trochanter on the ischial ramus after total hip arthroplasty: report of three cases. J Bone Joint Surg.

[4] Yanagishita CMA, Falótico GG, Rosário DAV, Pugina GG, Wever AAN, Takata ET (2015). Ischiofemoral Impingement - an etiology of hip pain: a case report. Rev Bras Ortop.

[5] Schubert T, Navez M, Galant C, Docquier P-L, Acid S, Lecouvet FE (2019). Femoral osteochondroma responsible for ischiofemoral impingement, bursitis, and secondary lipoma arborescens mimicking malignant transformation. Acta Radiol Open.

[6] Ali AM, Teh J, Whitwell D, Ostlere S (2013). Ischiofemoral impingement: a retrospective analysis of cases in a specialist orthopaedic centre over a four-year period. Hip Int.

[7] Torriani M, Souto SCL, Thomas BJ, Ouellette H, Bredella MA (2009). Ischiofemoral impingement syndrome: an entity with hip pain and abnormalities of the quadratus femoris muscle. AJR Am J Roentgenol.

[8] Kassarjian A (2008). Signal abnormalities in the quadratus femoris muscle: tear or impingement?. AJR Am J Roentgenol.

[9] Safran M, Ryu J (2014). Ischiofemoral impingement of the hip: a novel approach to treatment. Knee Surg Sports Traumatol Arthrosc.

[10] Sussman WI, Han E, Schuenke MD (2013). Quantitative assessment of the ischiofemoral space and evidence of degenerative changes in the quadratus femoris muscle. Surg Radiol Anat.

[11] Patti JW, Ouellette H, Bredella MA, Torriani M (2008). Impingement of lesser trochanter on ischium as a potential cause for hip pain. Skeletal Radiol.

[12] Wilson MD, Keene JS (2016). Treatment of ischiofemoral impingement: results of diagnostic injections and arthroscopic resection of the lesser trochanter. J Hip Preserv Surg.

[13] Kim W-J, Shin H-Y, Koo G-H, Park H-G, Ha Y-C, Park Y-H (2014). Ultrasound-guided prolotherapy with polydeoxyribonucleotide sodium in ischiofemoral impingement syndrome. Pain Pract.

[14] Lee S, Kim I, Lee SM, Lee J (2013). Ischiofemoral impingement syndrome. Ann Rehabil Med.

[15] Tosun Ö, Çay N, Bozkurt M, Arslan H (2012). Ischiofemoral impingement in an 11-year-old girl. Diagn Interv Radiol.

[16] Ganz R, Slongo T, Turchetto L, Massè A, Whitehead D, Leunig M (2013). The lesser trochanter as a cause of hip impingement: pathophysiology and treatment options. Hip Int.

[17] López-Sánchez MC, Armesto Pérez V, Montero Furelos LÁ, Vázquez-Rodríguez TR, Calvo Arrojo G, Díaz Román TM (2013). Ischiofemoral impingement: hip pain of infrequent cause. Reumatol Clin.

[18] Gómez-Hoyos J, Martin RL, Schröder R, Palmer IJ, Martin HD (2016). Accuracy of 2 clinical tests for ischiofemoral impingement in patients with posterior hip pain and endoscopically confirmed diagnosis. Arthroscopy.

[19] Peltola K, Heinonen OJ, Orava S, Mattila K (1999). Quadratus femoris muscle tear: an uncommon cause for radiating gluteal pain. Clin J Sport Med.

[20] Willick SE, Lazarus M, Press JM (2002). Quadratus femoris strain. Clin J Sport Med.

[21] Klinkert P, Porte RJ, de Rooij TP, de Vries AC (1997). Quadratus femoris tendinitis as a cause of groin pain. Br J Sports Med.

[22] Lu B, Deng H, Chen B, Zhao J (2019). The accuracy assessment of ultrasound for the diagnosis of ischiofemoral space–a validation study. J Xray Sci Technol.

[23] O’Brien SD, Bui-Mansfield LT (2007). MRI of quadratus femoris muscle tear: another cause of hip pain. Am J Roentgenol Am Roentgen Ray Soc.

[24] Singer AD, Subhawong TK, Jose J, Tresley J, Clifford PD (2015). Ischiofemoral impingement syndrome: a meta-analysis. Skeletal Radiol.

[25] Palczewski P, Sułkowska K, Świątkowski J, Kocoń H, Gołębiowski M (2015). Ischiofemoral impingement syndrome: a case report and a review of literature. Pol J Radiol.

[26] Taneja AK, Bredella MA, Torriani M (2013). Ischiofemoral impingement. Magn Reson Imaging Clin N Am.

[27] Kang M, Bang S-Y, Ryu JA, Gim S, Park E-S, Lee H (2016). A case of ischiofemoral impingement syndrome as a differential diagnosis of ankylosing spondylitis. J Rheum Dis Korean College of Rheumatol.

[28] Hernando MF, Cerezal L, Pérez-Carro L, Canga A, González RP (2016). Evaluation and management of ischiofemoral impingement: a pathophysiologic, radiologic, and therapeutic approach to a complex diagnosis. Skeletal Radiol.

[29] Gollwitzer H, Banke IJ, Schauwecker J, Gerdesmeyer L, Suren C (2017). How to address ischiofemoral impingement? Treatment algorithm and review of the literature. J Hip Preserv Surg.

[30] Nakano N, Shoman H, Khanduja V (2020). Treatment strategies for ischiofemoral impingement: a systematic review. Knee Surg Sports Traumatol Arthrosc.

[31] Truong WH, Murnaghan ML, Hopyan S, Kelley SP (2012). Ischioplasty for femoroischial impingement: a case report. JBJS Case Connector.

[32] Backer MW, Lee KS, Blankenbaker DG, Kijowski R, Keene JS (2014). Correlation of ultrasound-guided corticosteroid injection of the quadratus femoris with MRI findings of ischiofemoral impingement. AJR Am J Roentgenol.

[33] Howse EA, Mannava S, Tamam C, Martin HD, Bredella MA, Stubbs AJ (2014). Ischiofemoral space decompression through posterolateral approach: cutting block technique. Arthrosc Tech.

[34] Hatem MA, Palmer IJ, Martin HD (2015). Diagnosis and 2-year outcomes of endoscopic treatment for ischiofemoral impingement. Arthroscopy.

[35] Corrales R, Mediavilla I, Margalet E, Aramberri M, Murillo-González JA, Matsuda D (2018). Endoscopic lesser trochanter resection with refixation of the iliopsoas tendon for treatment of ischiofemoral impingement. Arthrosc Tech.

